# Supplementary material for: Transcriptomic Analysis of Gibberellin-Mediated Flower Opening Process in Tree Peony (Paeonia suffruticosa)
Source: Plants (Basel). 2025 Mar 23;14(7):1002. doi: 10.3390/plants14071002 (PMC11990408; doi:10.3390/plants14071002)
Supplement: Supplementary file 1 [file plants-14-01002-s001.zip › plants-3523508-supplementary.pdf]

Supplementary Figures

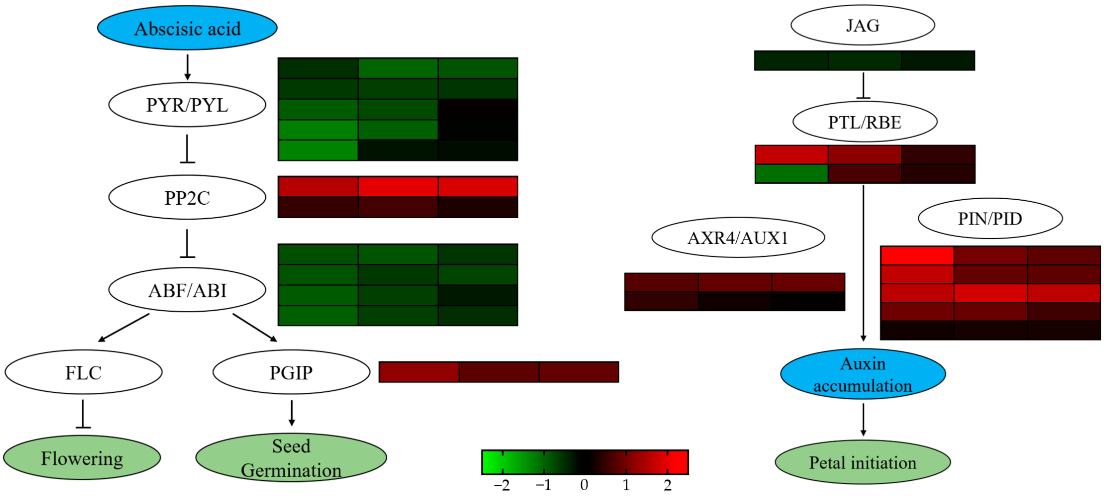

Supplementary Figure S1. Comparative analysis of DEGs involved in different signaling pathways under exogenous hormone treatments. The heatmap of plant hormone signal transduction-related DEGs under different treatments. Red meant the up-regulating and green meant the down-regulating. FDR < 0.05, $P < 0.05$ and |fold change| ≥ 2.

Supplementary Tables

Supplementary Table S1. Base Composition Statistics

Sample	RawData(bp)	CleanData(bp)	AF_Q20(%)	AF_Q30(%)	AF_GC(%)
CK-1	7041956100	6990038557	6824872906 (97.64%)	6508328262 (93.11%)	3138672837 (44.90%)
CK-2	8675103300	8597072160	8375757847 (97.43%)	7969466177 (92.70%)	3834652506 (44.60%)
CK-3	7679098200	7613419491	7390330104 (97.07%)	6996039864 (91.89%)	3408443120 (44.77%)
800-1	6863286000	6807036152	6636203992 (97.49%)	6312236194 (92.73%)	3038705807 (44.64%)
800-2	8397848100	8335396243	8128661121 (97.52%)	7741667600 (92.88%)	3725836679 (44.70%)
800-3	7984318200	7908910373	7723756928 (97.66%)	7367169707 (93.15%)	3782579944 (47.83%)
900-1	6754659900	6701666463	6539030436 (97.57%)	6231826951 (92.99%)	3023217511 (45.11%)
900-2	7473513300	7406239352	7198823033 (97.20%)	6826974767 (92.18%)	3291160650 (44.44%)
900-3	8165767800	8093900306	7878537674 (97.34%)	7482842310 (92.45%)	3587101182 (44.32%)
1000-1	7401948900	7341777694	7171213815 (97.68%)	6840373350 (93.17%)	3523700603 (48.00%)
1000-2	7410138900	7354247681	7157019045 (97.32%)	6798037491 (92.44%)	3286346758 (44.69%)
1000-3	7119531300	7062038218	6873701472 (97.33%)	6528707448 (92.45%)	3169402358 (44.88%)

Supplementary Table S1. Base Composition Statistics.

Supplementary Table S2. Annotation Statistics of Unigenes Against Four Databases

Total Unigenes	Nr	KEGG	KOG	SwissProt	Annotation genes	Without annotation gene
104888	46207	44272	25078	29063	47018	57870

Supplementary Table S2. Annotation Statistics of Unigenes Against Four Databases.
